# Ferroptosis: A novel therapeutic strategy and mechanism of action in glioma

**DOI:** 10.3389/fonc.2022.947530

**Published:** 2022-09-15

**Authors:** Gaosen Zhang, Yi Fang, Xiang Li, Zhen Zhang

**Affiliations:** Department of Ultrasound, First Affiliated Hospital of China Medical University, Shenyang, China

**Keywords:** ferroptosis, glioma, GPx4, system xc-, ncRNA

## Abstract

Glioma is the most common malignant tumor of the central nervous system and resistance is easily developed to chemotherapy drugs during the treatment process, resulting in high mortality and short survival in glioma patients. Novel therapeutic approaches are urgently needed to improve the therapeutic efficacy of chemotherapeutic drugs and to improve the prognosis of patients with glioma. Ferroptosis is a novel regulatory cell death mechanism that plays a key role in cancer, neurodegenerative diseases, and other diseases. Studies have found that ferroptosis-related regulators are closely related to the survival of patients with glioma, and induction of ferroptosis can improve glioma resistance to chemotherapy drugs. Therefore, induction of tumor cell ferroptosis may be an effective therapeutic strategy for glioma. This review summarizes the relevant mechanisms of ferroptosis, systematically summarizes the key role of ferroptosis in the treatment of glioma and outlines the relationship between ferroptosis-related ncRNAs and the progression of glioma.

## Introduction

Malignant tumors of the central nervous system are one of the most common types of cancer in humans and the incidence in the population is increasing year by year (1). Glioma is the most common primary malignant tumor of the adult central nervous system (2), accounting for 80% of all tumors (1). According to the latest WHO classification criteria (3), the pathological types of gliomas are divided into low-grade gliomas (LGG, grades 1–2) and high-grade gliomas (HGG, grades 3–4). WHO grade 4 glioblastoma (GBM) has the worst prognosis (4), with a median overall survival (OS) of only 12–17 months (5). At present, the treatment of glioma involves surgery, supplemented by radiotherapy, or a combination of radiotherapy and chemotherapy. LGG uses adjuvant chemotherapy with procarbazine, lomustine, and vincristine (6), and the survival time is significantly prolonged (7). Although high-grade gliomas are usually treated with temozolomide (TMZ) after surgery, the STUPP radiotherapy and chemotherapy regimen has prolonged the survival of patients to some extent (8), but the prognosis of some patients is still poor (9). This may be due to the limitations of glioma surgery methods, which do not allow complete separation of lesions from normal brain tissue, and insufficient tumor vascularization due to the existence of the blood-brain barrier and rapid proliferation of tumor cells (10). It is difficult to reach the tumor through blood circulation and achieve sufficient concentration to achieve localized function. Furthermore, HGG cells, especially GBM cells, exhibit extreme invasiveness (11) and heterogeneity (12). Therefore, treatment with a single chemotherapeutic agent can make glioma cells resistant (13), further complicating their heterogeneity and leading to glioma recurrence (14).

Ferroptosis is a novel iron-dependent regulatory cell death (RCD) method proposed by Dixon et al. (15). A large number of studies have shown that the role of ferroptosis in tumor therapy is particularly important (16–20). By inducing ferroptosis, tumor cell growth, migration, and invasion can be inhibited, achieving the purpose of tumor therapy (17). Drug resistance of tumor cells during chemotherapy can be reversed by inducing ferroptosis (18). After induction of ferroptosis, it can spread among surrounding cells, increasing the antitumor effect of chemotherapeutic drugs (19) and the sensitivity of tumor cells to chemoradiotherapy drugs (20). These findings provide new insight into the treatment of drug-resistant tumors. Recent studies have found that noncoding RNAs (ncRNAs) also play a key role in the ferroptosis of tumor cells (21, 22). ncRNAs such as microRNAs (miRNAs), long ncRNAs (lncRNAs), and circular RNAs (circRNAs) are widely involved in iron metabolism, ROS metabolism, and ferroptosis-related amino acid metabolism in tumor cells (22). Glioma cells have extensive heterogeneity and are prone to drug resistance to chemotherapeutic drugs, resulting in an unsatisfactory patient prognosis (23). A study has found that the ferroptosis inducer erastin can enhance the sensitivity of GBM cells to TMZ (24). Therefore, this new strategy to induce ferroptosis in tumor cells may have great potential in the treatment of glioma. Current research focuses on how to reduce drug resistance in glioma chemotherapy and deepens the mechanism of classical ferroptosis-inducing pathways such as GPX4 in glioma. Ferroptosis-related ncRNAs and nanoparticle therapy targeting ferroptosis in gliomas have received increasing attention. This review summarizes the mechanism of ferroptosis in tumors and the research progress of ferroptosis in the treatment of glioma, as well as the key role of ferroptosis-related ncRNAs, and ultimately the potential clinical value of ferroptosis in personalized treatment regimens for glioma.

## Mechanisms of ferroptosis in tumors

Ferroptosis is fundamentally different from apoptosis, pyroptosis, autophagy, and other cell death methods, and shares no similarities in terms of morphology, biochemistry, or genetics (25). Ferroptosis is characterized by the availability of redox-active iron (26) the loss of lipid peroxide repair capacity by the phospholipid hydroperoxidase GPX4 (27), and oxidation of polyunsaturated fatty acid (PUFA)-containing phospholipids (28). Ferroptosis is triggered in tumor cells by cysteine (Cys) depletion or by inhibition of glutathione peroxidase 4 (GPX4) (29). Subcellular structural changes are manifested by the reduction or disappearance of mitochondrial cristae in tumor cells and the destruction of the inner and outer mitochondrial membranes (30). Below we provide an overview of its mechanism around important features of ferroptosis in tumor cells.

### Disruption of iron homeostasis

Iron is the basis of tumor cell proliferation, metabolism, invasion, and disruption of the intracellular environment and cytoplasmic iron homeostasis is a key regulator that induces ferroptosis (31). Tumor cells are more proliferative than normal cells and have a greater need for iron (32). The excess free iron produced by the Fenton reaction (33) or iron-containing lipoxygenase (34) oxidizes PUFA on the cell membrane to increase the formation of lipid ROS. Hydroxyl free radicals are the most active substances in ROS, which can trigger the production of PUFAs in membrane lipids. peroxidation, leading to ferroptosis in cells (35). Therefore, an increase in Fe^2+^ content in cells increases the sensitivity of cells to ferroptosis, and iron chelators such as deferoxamine can inhibit ferroptosis by chelating Fe^2+^ in cells to interfere with the production of oxidized lipids (27). Transferrin (Tf) usually binds to the transferrin receptor (TfR1) to transport iron from the intracellular environment into cells in the form of iron-transferrin complexes, a process that is important in ovarian cancer (36), sarcoma (37), and other tumors were significantly up-regulated. TfR1-mediated downregulation of the iron-transferrin complex reduces cellular iron uptake, thus inhibiting ferroptosis (38). Furthermore, heat shock protein HSPB1 and phosphorylated HSPB1 also reduce iron uptake in cells from the internal environment (39), reducing the sensitivity of cells to ferroptosis. Endogenous iron is released into the cytoplasm by lysosomes under the influence of acidic conditions, and this process depends on the phagocytosis of ferritin by nuclear receptor coactivator 4 (NCOA4) (40). Knockdown of NCOA4 inhibits ferritin degradation, reduces cytoplasmic iron, and up-regulates ferritin heavy chain (FTH1) expression, thus inhibiting erastin-induced ferroptosis (41). Furthermore, the pentaspanin membrane glycoprotein prominin-2 promotes the formation of exosomes and transports cytoplasmic iron out of the cell (42). CDGSH iron-sulfur cluster domain 1 (CISD1) (43) and the iron-sulfur cluster biosynthesis enzyme (NFS1) (44) reduce cell susceptibility to ferroptosis by uptake of cytoplasmic iron.

### Lipid peroxidation

Human cell membranes are rich in PUFA-acylated glycerophospholipids and have a wide variability (45) and the scavenging of peroxidized PUFAs can inhibit ferroptosis (46). PUFAs are very sensitive to free radical or enzyme-mediated oxidation (47), and peroxidized PUFAs bind to the glycerophospholipids of the cell membrane and participate in tumor cell ferroptosis and cause cell membrane destruction (48). Excess GSH accumulation due to down-regulation of GPX4 is a molecular mechanism leading to lipid peroxidation (49). GPX4 can reduce lipid hydroperoxides to lipid alcohols to avoid lipid peroxidation (50). Rapamycin complex 1 (mTORC1) upregulates GPX4 expression and inhibits membrane lipid peroxidation (51). The ferroptosis activator RSL3 can induce ferroptosis in tumor cells by silencing or inhibiting GPX4 expression (26). System Xc^-^ inhibition is another molecular mechanism leading to lipid peroxidation (49). System Xc^-^ is a glutamate (Glu)/cystine antiporter composed of the solute carrier family 3 member 2 (SLC3A2) and the solute carrier family 7 member 11 (SLC7A11). The ferroptosis activator erastin inhibits system Xc^-^ blocking the entry of cystine into cells, and the lack of intracellular Cys leads to a decrease in GSH and a decrease in GPX4 activity (52). The tumor suppressor protein BRCA1-associated protein 1 (BAP1) inhibits SLC7A11 expression in a deubiquitinating-dependent manner and induces lipid peroxidation to promote ferroptosis (53). Membrane-associated progesterone receptor component 1 (PGRMC1) inhibits SLC7A11 through autophagic degradation of lipids and induces ferroptosis in paclitaxel-resistant tumor cells (54). NADPH oxidase (NOX) and the tumor suppressor gene p53 (especially the acetylation-deficient mutant p53-3KR) also inhibit SLC7A11 (55). A recent study found that the homology of m6A reader YT521-B containing 2 (YTHDC2) can induce ferroptosis in lung adenocarcinoma cells by inhibiting SLC7A11 (56). Moreover, YTHDC2 suppressed SLC3A2 by inhibiting Homeobox A13 (HOXA13) indirectly (57) and was also found to affect system Xc^-^ function in lung adenocarcinoma cells.

### Accumulation of reactive oxygen species

Reactive oxygen species (ROS) are closely related to the proliferation and death of tumor cells. A certain amount of ROS can promote tumor signal transduction and promote tumor cell proliferation, growth, and adaptation to hypoxia. However, excessive ROS accumulation promotes antitumor signaling, triggers oxidative stress, and induces cell death (58). The content of ROS in tumor cells is higher than in normal cells, and the neuronal redox sensing channel TRPA1 can improve the defense ability of tumor cells against ROS (59). The continuous accumulation of ROS in tumor cells eventually leads to the disappearance of mitochondrial ridges and the destruction of mitochondrial membranes, leading to ferroptosis (60). GPX4 (61), vitamin E (α-tocopherol), and coenzyme Q 10 (CoQ 10) can reduce membrane lipid ROS (62). The Fenton reaction, NADPH-dependent lipid peroxidation, GSH depletion, and decreased GPX4 activity can all promote ROS accumulation in tumor cells (63), which induces ferroptosis in tumor cells.

These studies revealed the complex regulatory mechanism of ferroptosis in tumor cells, which can promote or inhibit ferroptosis by regulating key regulators of ferroptosis. We list the key regulators related to ferroptosis in glioma and their regulatory mechanisms and further explore the application of ferroptosis in the treatment of glioma.

## Mechanisms of ferroptosis in glioma

**Figure 1:**
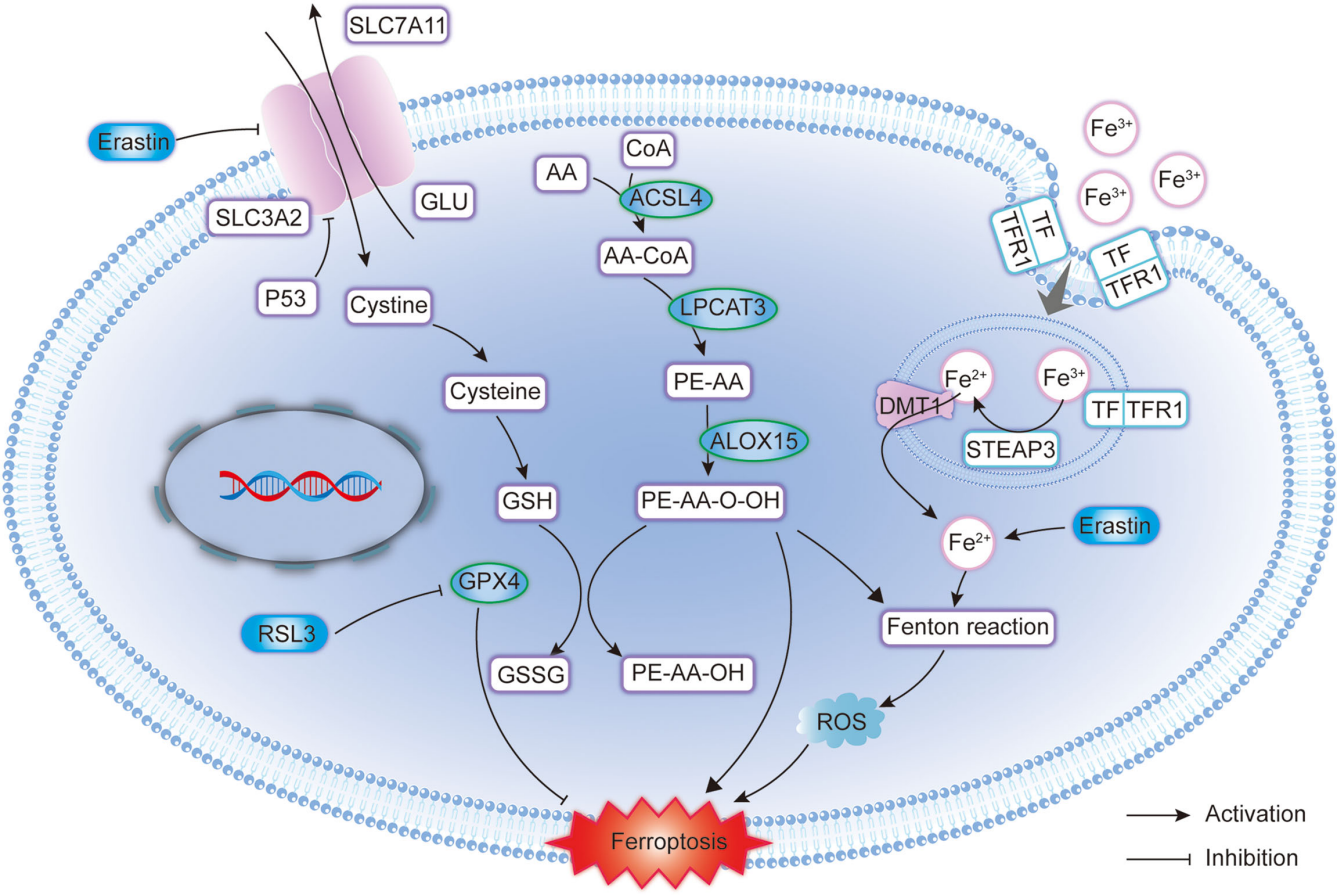
Molecular pattern diagram of ferroptosis in glioma. GPX4, Glutathione Peroxidase 4; SLC7A11, Solute carrier family 7 membrane 11; SLC3A2, Solute carrier family 3 membrane 2; GSH, Glutathione; GSSG, Glutathione disulphide; ACSL4, Acyl-CoA synthetase long-chain family member 4; ROS, Reactive oxygen species; DMT1, Divalent metal transporter 1; LPCAT3, Lysophosphatidylcholine acyltransferase 3; STEAP3, Six-transmembrane epithelial antigen of the prostate 3; TFR1, Transferrin receptor 1; TF, Transferrin; ALOX15, Arachidonate 15-lipoxygenase.

Glioma cells undergo marked metabolic reprogramming (such as the Warburg effect) and GBM cell membrane lipid species are highly cell-type specific, with lipid metabolism involved in the occurrence and progression of GBM (64). The reprogramming of lipid metabolism is affected by the efficiency of acetyl-CoA (65) and isocitrate dehydrogenase (IDH) (66). In particular, the IDH mutation is particularly important for the prognosis of glioma. The IDH mutation inhibits the function of the wild-type IDH product α-ketoglutarate by abnormally producing 2-hydroxyglutarate (2-HG), thus affecting the lipid metabolism of glioma cells (67). Ivanov et al. (68) fed glioma-transplanted rats with a prolonged diet including iron-containing water and found that it promoted the growth of gliomas in rats and improved the effects of radiotherapy, which disappeared after the injection of deferoxamine. Another study (69) suggested that iron and iron metabolism could affect the prognosis of patients with glioma, and the study also found that key regulators of ferroptosis were also important in neuronal function. Alim (70) found that selenium could inhibit GPX4-dependent ferroptosis in neuronal cells. GPX4 depletion leads to neurodegeneration *in vivo* and *in vitro* (71). Below, we focus on the key regulators of ferroptosis and elaborate on the mechanisms of ferroptosis in glioma (**Figure 1**).

### GPX4

GPX4 is a selenoprotein that belongs to the glutathione peroxidase family (GPX1-8) and is a key regulator in ferroptosis (72). Normally active GPX4 can reduce lipid peroxide (LPO) to alcohol or reduce intracellular H_2_O_2_ to water to avoid or reduce cell membrane lipid peroxidation. Reduced or inactivated GPX4 activity leads to an excessive accumulation of ROS on membrane lipids that leads to ferroptosis (73). Fragile X-related protein-1 RNA binding protein (FXR1) in glioma cells can bind to GPX4 mRNA and up-regulate GPX4 expression (74). Nrf2 can also inhibit ferroptosis by up-regulating GPX4 expression in glioma cells (75). Conversely, activation of the p38 and ERK pathways in GBM decreased the levels of GPX4 protein (76). Helena Kram et al. (77) performed immunohistochemistry on sample pairs of primary and relapse GBM of 24 patients who had received standard adjuvant treatment with radiochemotherapy. They found that the expression of GPX4 decreased significantly during tumor relapse. this study shows that recurrent tumors have a higher vulnerability to ferroptosis.

### System Xc^-^

System Xc^-^ consists of two parts, SLC7A11 and SLC3A2, and its main function is to transport extracellular cystine into cells and reverse glutamate (Glu) transport. A study has found that SLC7A11 is up regulated in a variety of tumor cells, promotes glutathione (GSH) synthesis to inhibit damage from oxidative stress to tumor cells, and is negatively correlated with the median OS of patients (78). System Xc^-^ also plays a significant role in ferroptosis in gliomas. The proper functioning of System Xc^-^ function is critical for neuronal signaling (79). Activating transcription factor 4 (ATF4) can increase neovascularization within gliomas and shape neovascularization in a SLC7A11-dependent manner (80). The expression of p53 is deregulated in GBM, and studies have found that the expression of p53 and SLC7A11 is negatively correlated in glioma cells (81) and that p53 inhibits the expression of the SLC7A11 gene (82). Furthermore, p62 binds to p53 and inhibits p53 ubiquitination in GBM. The canonical p62-mediated Nrf2 activation pathway plays an important role in the regulation of ferroptosis in wild-type GBM p53 and inhibits ferroptosis by upregulating the expression of SLC7A11.

In GBM p53 mutants, the strong interaction of p62 with mutant p53/Nrf2 enhances the inhibitory effect of mutant p53 on Nrf2, thus reversing the classical p62-mediated Nrf2 activation pathway (83). However, one study found that the tumor stem cell marker CD44 inhibited ferroptosis in tumor cells in a manner dependent on the deubiquitinase OTUB1, and overexpression of CD44 improved the stability of the SLC7A11 protein by promoting the interaction between SLC7A11 and OTUB1 (84). High expression of OTUB1 was also found in clinical samples of glioma and was positively correlated with SLC7A11 expression (85). Furthermore, the NF-κB pathway activator protein of the NF-B pathway promotes the splicing and maturation of SLC7A11 mRNA by binding to m6A, thus upregulating the expression of SLC7A11 and inhibiting ferroptosis in GBM cells (86).

### Other regulators

MDM2 and MDMX are negative regulators of p53, and inhibition of MDM2 or MDMX increases ferroptosis suppressing protein 1 (FSP1) expression in glioma cells. The MDM2-MDMX complex regulates lipid peroxidation and promotes ferroptosis in glioma cells by altering the activity of PPARα (87). Knockdown of COPZ1 in GBM cells leads to increased expression of nuclear receptor coactivator 4 (NCOA4), and inhibition of FTH1 leads to ferritin degradation, resulting in excessive accumulation of intracellular Fe^2+^, leading to ferroptosis (88). Inhibition of the expression of the matrix remodeling-related protein MXRA8 can also up-regulate NCOA4 and down-regulate FTH1 expression, and MXRA8 is positively correlated with the macrophage marker CSF1R. One study co-cultured glioma cell with M2 macrophages and found that MXRA8 knockdown in glioma cells attenuated the infiltration of M2 macrophages, while the addition of Fer-1 restored the infiltration of M2 macrophages (89). The deletion of NCOA4 can inhibit the reduction in the level of the FTH1 protein caused by cystine deprivation, and cystine deprivation simultaneously induces the accumulation of light chain 3 (LC3)-II protein associated with microtubules, enhances ferritin phagocytosis, and then promotes ferroptosis in GBM cells (90). The study (91) has also found that phosphorylation of heat shock protein 27 (HSP27) in GBM cells can resist erastin-induced ferroptosis, while down-regulation of HSP27 promotes erastin-induced ferroptosis and can function as a negative regulator of ferroptosis.

The above studies confirmed the feasibility of treating glioma with classical ferroptosis regulators and mechanisms such as GPX4, SLC7A11, and FSP1. Below, we focus on the key regulators of ferroptosis in the above-mentioned gliomas and describe current ferroptosis-related glioma treatment strategies.

## Ferroptosis in glioma treatment

### Regulation of GPX4

Studies have found that ibuprofen can induce ferroptosis in glioma cells by down-regulating GPX4 expression in Nrf2-regulated cells (75). GPX4 is a key regulator for dual artemisinin (DHA)-induced ferroptosis in GBM cells (92). DHA induces endoplasmic reticulum (ER) stress in glioma cells, leading to upregulation of ATF4 and protein kinase R-like ER kinase (PERK). ATF4 induces the overexpression of heat shock protein family A (Hsp70) member 5 (HSPA5) and increases GPX4 expression and activity. GPX4 neutralized DHA induces lipid peroxidation, thus protecting glioma cells from ferroptosis (93). Curcumin analogs induce androgen receptor (AR) ubiquitination to inhibit GPX4 activity, thus promoting ferroptosis and reducing resistance to TMZ in GBM cells (94). Studies have found that FXR1 expression is increased in TMZ-resistant glioma cells, and targeted inhibition of FXR1-GPX4 can reduce the drug resistance of TMZ-resistant glioma cells (74). Dihydrotanshinone I (DHI) increases ACSL4 expression in glioma cells and down-regulates GPX4 to inhibit glioma cell proliferation (95). The anti-malaria drug artesunate (ART) induces ferroptosis in GBM cells by regulating the p38 MAPK and ERK signaling pathways and reducing the level of GPX4 protein (76). Although gastrodin reduces the level of malondialdehyde (MDA) in rat glioma cells, which in turn increases GPX4 activity and inhibited ferroptosis in rat glioma cells (96), plumbagin induces GPX4 degradation in glioma cells *via* the lysosomal pathway and leads to GPX4-dependent cell death (97).

RSL3, a small molecule compound that can target GPX4, induces glycolytic dysfunction and autophagy-dependent ferroptosis in glioma cells (98). While down regulating GPX4, RSL3 also activates the nuclear factor kappa-B (NF-κB) pathway to induce ferroptosis in GBM cells. However, the study found that knockdown of GPX4 alone did not effectively induce ferroptosis in glioma cells. NF-κB pathway activation combined with GPX4 silencing induces ferroptosis and inhibits glioma growth and recurrence (99). Ferroptosis activators can inhibit GPX4 expression and synergize with radiotherapy, inducing ferroptosis in glioma cells without increasing DNA damage (100). Local chemotherapy is also a new direction in the treatment of glioma, increasing local chemotherapy drugs while minimizing the impact on normal cells, to inhibit tumor growth and recurrence. The study has reported on the use of gene therapy-based iron oxide nanoparticles (IONP) to deliver GPX4 small interfering RNA (siRNA) and cisplatin (Pt). Nanoparticles activate NADPH oxidase (NOX) to increase H_2_O_2_ levels while releasing si-GPX4 to inhibit GPX4 expression, causing excessive ROS accumulation and triggering ferroptosis in glioma cells (101). In one study, paclitaxel-loaded iron oxide nanoparticles (IONP@PTX) enhanced the expression of autophagy-related proteins Beclin1 and LC3II, inhibited the expression of p62 protein, and GPX4, and induced ferroptosis in GBM cells (102). Tumor immunity-related studies have found that certain tumor lesions that occur during early tumor progression (i.e., ischemia) recruit neutrophils to sites of tissue damage. However, neutrophils can induce ferroptosis in GBM cells by regulating GPX4, creating a positive feedback loop that exacerbates the development of internal GBM necrosis (103).

### Regulation of SLC7A11

Studies have found that ibuprofen affects GPX4 expression in glioma cells by downregulating Nrf2 and at the same time inhibits the activity of SLC7A11 (75). Activation of the Nrf2-Keap1 pathway up-regulates SLC7A11 to release a large amount of Glu out of glioma cells, thereby affecting the tumor microenvironment, which may be related to the decreased survival rate of patients with glioma with high expression of SLC7A11 (104). Like ibuprofen, in addition to downregulating GPX4, plumbagin can significantly down-regulate SLC7A11 mRNA and protein levels in glioma cells and induce ferroptosis in glioma cells (97). As a first-line drug for chemotherapy with GBM in clinical practice, TMZ can affect GPX4 and reduces the activity of SLC7A11. Studies have found that gliomas with high expression of SLC7A11 are more sensitive to erastin-TMZ combination therapy and have better therapeutic effects (105). ATF4 is a key regulator in cellular metabolism and maintenance of oxidative homeostasis, and upregulation of SLC7A11 expression by ATF4 improves the resistance of gliomas to chemotherapeutic drugs such as TMZ (106). However, the latest study found that down-regulation of ATF4 in GBM cells inhibited CHAC1 expression and blocked sevoflurane (Sev)-induced ferroptosis (107). Activating transcription factor 3 (ATF3) in glioma can promote ferroptosis of glioma cells by upregulating NOX4 and SOD1 to produce H_2_O_2_ and promote the strychnine-induced accumulation of H_2_O_2_, and by downregulating SLC7A11 to prevent degradation of H_2_O_2_ (108). Pseudolaric acid B (PAB) increases the intracellular iron content in the glioma by upregulating the transferrin receptor, activating NOX4, and producing excess H_2_O_2_ and LPO. PAB blocks cystine supply through the p53-mediated SLC7A11 pathway, depleting intracellular GSH and further exacerbating H_2_O_2_ and LPO accumulation (109). RSL3 is a GPX4 inhibitor, and recent studies have found that RSL3 can down-regulate SLC7A11 expression by activating the NF-κB pathway (99).

### Regulation of other key regulators of ferroptosis

Differences in transferrin receptor (TfR) in normal human astrocytes (NHA) and GBM cell lines may be the key to DHA selective killing of tumor cells to induce ferroptosis (92). A team designed cRGD/Pt + DOX@GFNPs (RPDGs) nanoparticles to promote the simultaneous occurrence of apoptosis and ferroptosis by disrupting redox homeostasis in mouse GBM-resistant cells. Using the Fenton reaction of gallic acid (GA)/Fe^2+^ to catalyze nanoparticles in the intracellular environment, Pt (IV) depletes intracellular GSH and increases the accumulation of reactive oxygen species (ROS), thus inducing ferroptosis in GBM-resistant cells (110). Fe_3_O_4_-siPD-L1@M-_BV2_ increased Fe^2+^ accumulation in mouse GBM-resistant cells and significantly decreases the expression of programmed death-ligand 1 (PD-L1). Fe_3_O_4_-siPD-L1@M-_BV2_ also increases the ratio of effector T cells to regulatory T cells in drug-resistant GBM (111). Amentoflavone (AF) can not only induce autophagy in glioma cells by regulating the AMPK/mTOR pathway but is also associated with ferroptosis in gliomas. Knockdown of autophagy-related protein 7 (ATG7) was found to increase ferritin heavy chain 1 (FTH1) expression and inhibits AF-induced ferroptosis. It demonstrates that AF triggered ferroptosis in an autophagy-dependent manner, thereby suppressing glioma growth and recurrence (112). Another study found that siramesin combined with lapatinib mediates ferroptosis in glioma cells through iron release in lysosomes and protease degradation of HO-1 (113). Doranidazole and misonidazole can induce ferroptosis by blocking metabolic alterations in mitochondrial complex I and II of hypoxic glioma stem cells (GSC) that trigger responses to oxidative stress (114).

The above studies demonstrate the mechanism by which different drugs treat glioma by modulating key regulators of ferroptosis (**Table 1**). In addition, ferroptosis-related ncRNAs also have a certain influence on the treatment of glioma. Next, we will elaborate on ferroptosis-related ncRNAs.

**Table 1:** Summary of ferroptosis-associated agents.

Ferroptosis-associated agents	Mechanism	Function	Study
Ibuprofen	down-regulates GPX4 expression and inhibits the activity of SLC7A11	Induces ferroptosis	(75)
Dual artemisinin	up-regulates ATF4 induces the overexpression of HSPA5 and increases GPX4 expression and activity	Inhibits ferroptosis	(93)
Curcumin analog	induces AR ubiquitination to inhibit GPX4 activity	Induces ferroptosis	(94)
Dihydrotanshinone I	increases ACSL4 expression and down-regulates GPX4	Induces ferroptosis	(95)
Artesunate	down-regulates GPX4	Induces ferroptosis	(76)
Plumbagin	induces GPX4 degradation *via* the lysosomal pathway and down-regulates SLC7A11 mRNA and protein expression	Induces ferroptosis	(97)
RSL3	inhibits GPX4 and down-regulates SLC7A11 expression by activating the NF-κB pathway	Induces ferroptosis	(99)
IONP	activates NOX to increase H_2_O_2_ levels while releasing si-GPX4 to inhibit GPX4 expression	Induces ferroptosis	(101)
IONP@PTX	up-regulates the expression of autophagy-related proteins Beclin1 and LC3II, and inhibits the expression of p62 and GPX4	Induces ferroptosis	(102)
Temozolomide	down-regulates GPX4 and reduces the activity of SLC7A11	Induces ferroptosis	(105)
Amentoflavone	induces autophagy by regulating the AMPK/mTOR pathway, and down-regulates FTH1 expression	Induces ferroptosis	(112)
Siramesin and lapatinib	*Via* iron release in lysosomes and protease degradation of HO-1	Induces ferroptosis	(113)

## Ferroptosis-related ncRNAs in glioma treatment

**Figure 2:**
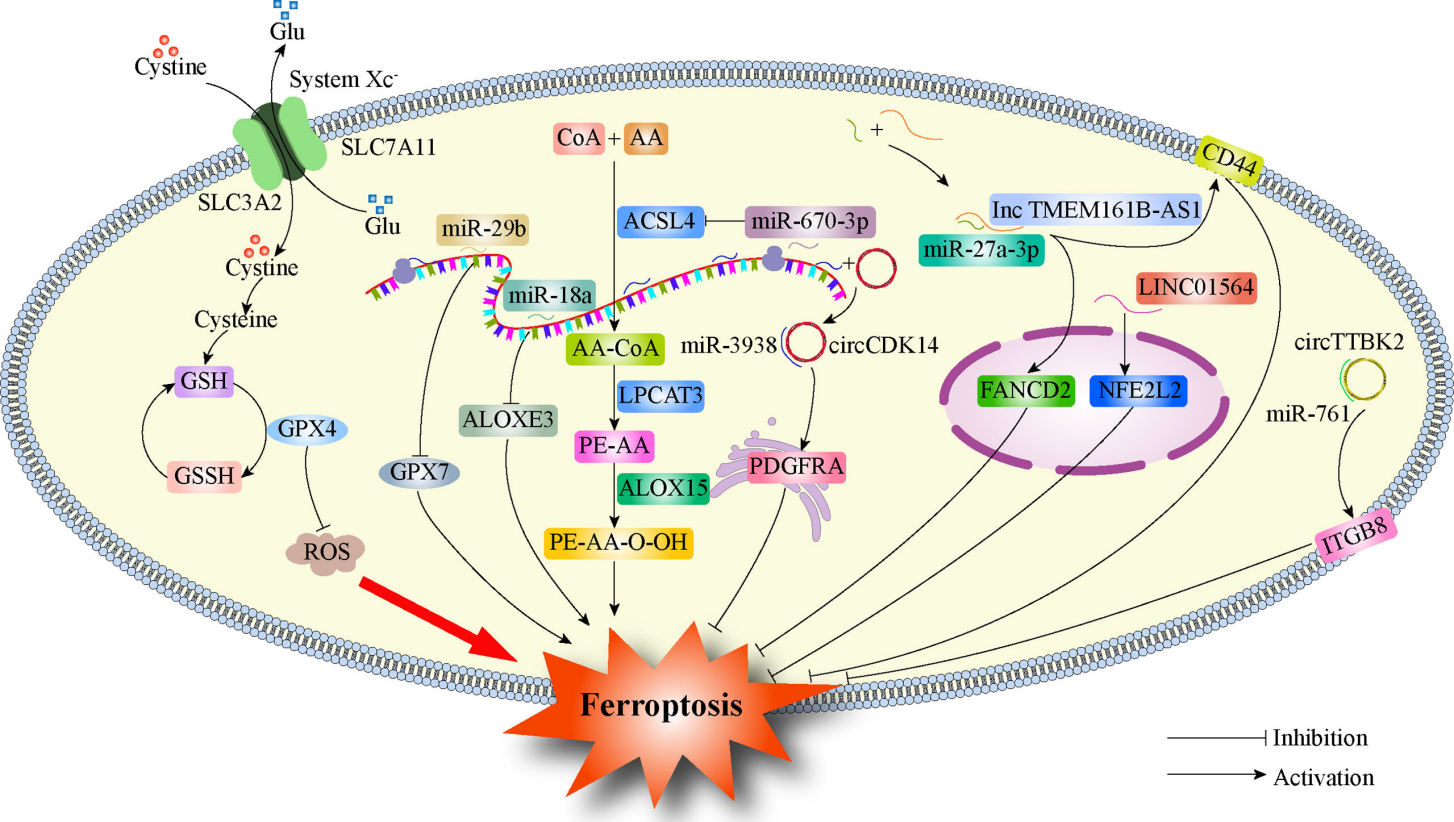
Regulation of ferroptosis by ncRNAs in glioma. GPX4, Glutathione Peroxidase 4; SLC7A11, Solute carrier family 7 membrane 11; SLC3A2, Solute carrier family 3 membrane 2; GPX7, Glutathione Peroxidase 7; ACSL4, Acyl-CoA synthetase long-chain family member 4; ALOXE3, Arachidonate lipoxygenase 3; PDGFRA, Platelet-derived growth factor receptor; FANCD2, FA Complementation Group D2; CD44, Cluster of differentiation 44; NFE2L2, Nuclear factor erythroid 2-like 2; ITGB8, Integrin subunit beta 8.

Ferroptosis and ncRNAs are closely related to tumors (115). Among ncRNAs, miRNAs, lncRNAs, and circRNAs are all involved in the potential regulatory mechanisms of tumor ferroptosis (116). ncRNAs can regulate the protein levels of ferroptosis-related genes (117), influence the expression of mRNA of ferroptosis-related genes (118), lead to modification of m6A (117), and control epigenetic activity (119). ncRNAs induce ferroptosis by regulating cellular iron metabolism, ROS metabolism, and lipid metabolism. Recent studies have found that ncRNAs also play a key role in glioma ferroptosis (**Figure 2**). GPX7 is a member of the glutathione peroxidase family (GPX), and miR-29b can inhibit GPX7 expression, thus improving the sensitivity of glioma cells to erastin-induced ferroptosis (120). miR-670-3p inhibited GBM cell ferroptosis by downregulating ACSL4 expression, while the miR-670-3p inhibitor increased the antitumor effect of TMZ (121). miR-18a inhibited ferroptosis related to p53-SLC7A11 in GBM cells by down-regulating the expression of ALOXE3 (122). The lncRNA TMEM161B-AS1 increases FANCD2 and CD44 expression by adsorbing hsa-miR-27a-3p, inhibits apoptosis and ferroptosis, but reduces the resistance to TMZ in GBM cells (123). LINC01564 has the opposite effect, inhibiting ferroptosis by upregulating NFE2L2 expression and enhancing glioma cell resistance to TMZ (124). Circular RNA CDK14 upregulates the expression of the PDGFRA oncogene in GBM cells by adsorbing miR-3938 and reducing the sensitivity of GBM to erastin-induced ferroptosis (125). The circRNAs TTBK2 and ITGB8 are highly expressed in glioma tissues and cells, and TTBK2 can inhibit ferroptosis in glioma cells by up-regulating ITGB8 by adsorbing miR-761 (126). **Table 2** summarizes the findings from recent studies as these results suggest that ncRNAs play a key role in ferroptosis in gliomas and may become new therapeutic targets for gliomas.

**Table 2:** The regulatory role of ferroptosis-related ncRNAs in glioma progression.

NcRNA	Cell Lines	Mechanism	Function	Study
miR-29b	U87 T98G LN229 A172	Target GPX7	Induces ferroptosis and enhances glioma cell sensitivity to erastin-induced ferroptosis	(120)
miR-670-3p	U87MG A172	Target ACSL4	Inhibits ferroptosis	(121)
miR-18a	U87MG U251	Target ALOXE3	Inhibits ferroptosis and promote migration	(122)
lncRNA TMEM161B-AS1	U87MG U251	Sponge with mir-27a-3p and upregulate the expression of FANCD2 and CD44	Inhibits ferroptosis	(123)
LINC01564	LN229/TMZ U251/TMZ	Upregulate the expression of NFE2L2	Inhibits ferroptosis and promote TMZ resistance	(124)
circ CDK14	HBE SF126 U251 U87	Sponge with mir-3938 and upregulate the expression of PDGFRA	Inhibits ferroptosis	(125)
circ TTBK2	LN229 U251 NHA	Sponge with mir-761 and upregulate the expression of ITGB8	Inhibits ferroptosis	(126)

## Conclusions and future prospects

The heterogeneity of glioma cells alters the sensitivity of gliomas to different chemotherapeutic drugs. The use of a certain chemotherapeutic drug alone in the treatment process cannot achieve the expected desired effect, and new treatment methods are needed to supplement it. The induction of ferroptosis in tumor cells has attracted increasing attention as a new strategy for the treatment of glioma. Previous studies have preliminarily explored the mechanism of ferroptosis in glioma and found that improved iron metabolism and resistance to lipid peroxidation are prevalent in glioma cells. However, recurrent GBM showed high sensitivity to ferroptosis. These studies demonstrate ferroptosis as a new option for glioma treatment in the face of tumor resistance and recurrence. Protein molecules such as GPX4 and System Xc^-^ and ferroptosis-related ncRNAs have become important targets for glioma therapy. Current studies have demonstrated the value and potential of treating glioma through the canonical pathway and factors of ferroptosis. However, GPX4 is an essential gene in mammals, and whether drugs that inhibit GPX4 to treat tumors will bring unbearable side effects to glioma patients remains to be further studied. Therefore, it is particularly important to explore non-canonical pathways of ferroptosis in the treatment of glioma, such as the treatment of glioma through the HO-1 pathway. In addition, the current study does not involve the treatment of recurrent glioma, and ferroptosis-related ncRNA research and molecular therapy are also in their infancy. Therefore, it is particularly important to study ferroptosis-related mechanisms in greater detail in glioma and to explore ferroptosis-related ncRNAs, nanoparticles, and exosomes. Currently, the clinical trials investigating ferroptosis applied to the treatment of glioma are still incomplete. Inducing ferroptosis to destroy tumor cells and reducing damage to normal cells of the central nervous system is the key to promoting the clinical translation of research findings. In conclusion, as a complement to current therapeutic approaches, ferroptosis has immense potential in the treatment and prognosis of glioma.

## Author contributions

GZ and YF participated in the conception and designed this review. ZZ provided administrative support. GZ wrote the manuscript. YF and XL revised the manuscript. All authors contributed to this article and approved the submitted version.

## Funding

This research was supported by the National Natural Science Foundation of China (No.81471809; No.81971639).

## Conflict of interest

The authors declare that the research was conducted in the absence of any commercial or financial relationships that could be construed as a potential conflict of interest.

## Publisher’s note

All claims expressed in this article are solely those of the authors and do not necessarily represent those of their affiliated organizations, or those of the publisher, the editors and the reviewers. Any product that may be evaluated in this article, or claim that may be made by its manufacturer, is not guaranteed or endorsed by the publisher.
